# A routine quality assurance test for CT automatic exposure control systems

**DOI:** 10.1120/jacmp.v17i4.6165

**Published:** 2016-07-08

**Authors:** Gareth R. Iball, Alexis C. Moore, Elizabeth J. Crawford

**Affiliations:** ^1^ Department of Medical Physics & Engineering Leeds General Infirmary Leeds LS1 3EX UK

**Keywords:** computed tomography, quality assurance, automatic exposure control, dosimetry, image quality

## Abstract

The study purpose was to develop and validate a quality assurance test for CT automatic exposure control (AEC) systems based on a set of nested polymethylmethacrylate CTDI phantoms. The test phantom was created by offsetting the 16 cm head phantom within the 32 cm body annulus, thus creating a three part phantom. This was scanned at all acceptance, routine, and some nonroutine quality assurance visits over a period of 45 months, resulting in 115 separate AEC tests on scanners from four manufacturers. For each scan the longitudinal mA modulation pattern was generated and measurements of image noise were made in two annular regions of interest. The scanner displayed CTDIvol and DLP were also recorded. The impact of a range of AEC configurations on dose and image quality were assessed at acceptance testing. For systems that were tested more than once, the percentage of CTDIvol values exceeding 5%, 10%, and 15% deviation from baseline was 23.4%, 12.6%, and 8.1% respectively. Similarly, for the image noise data, deviations greater than 2%, 5%, and 10% from baseline were 26.5%, 5.9%, and 2%, respectively. The majority of CTDIvol and noise deviations greater than 15% and 5%, respectively, could be explained by incorrect phantom setup or protocol selection. Barring these results, CTDIvol deviations of greater than 15% from baseline were found in 0.9% of tests and noise deviations greater than 5% from baseline were found in 1% of tests. The phantom was shown to be sensitive to changes in AEC setup, including the use of 3D, longitudinal or rotational tube current modulation. This test methodology allows for continuing performance assessment of CT AEC systems, and we recommend that this test should become part of routine CT quality assurance programs. Tolerances of ±15% for CTDIvol and ±5% for image noise relative to baseline values should be used.

PACS number(s): 87.57.Q‐

## I. INTRODUCTION

Computed tomography (CT) automatic exposure control (AEC) systems were first introduced in the mid‐1990s, but have become an integral part of all modern CT systems, including those used in Nuclear Medicine and Radiotherapy.^1^, ^2^, ^3^, ^4^, ^5^ The first such systems were known as automatic tube current modulation systems (ATCM), as they solely adjusted the X‐ray tube current during the rotation of the X‐ray tube and detector system around the patient to account for angular variations in patient attenuation. It was not until the introduction of 3D tube current modulation systems (i.e., those that adjust for overall patient size and both longitudinal and rotational variations in attenuation) that CT scanners could be considered to employ true AEC systems.

Routine quality assurance (QA) tests for radiographic, mammographic, and fluoroscopic AEC systems are well established and documented in the literature.^6^, ^7^, ^8^, ^9^, ^10^, ^11^, ^12^ In addition, standard AEC test objects, generally consisting of acrylic blocks, copper sheets or water phantoms, are also available for such X‐ray systems.^6^, ^7^, ^8^, ^9^, ^10^, ^11^, ^12^ However, there is currently no standardized QA test for CT AEC systems. Since the CT AEC system determines the appropriate dose for an examination on a patient‐by‐patient basis, the AEC system is a major component in the determination of patient dose and image quality in clinical CT scanning. It is therefore important to test the functionality of the AEC system, both at acceptance testing and on a routine basis.

The Nordic guidance on CT QA^(13)^ lists CT AEC testing as an optional test and it is suggested that a cone‐shaped or anthropomorphic phantom could be used to assess the functionality of the AEC system, via noise measurements along the length of the phantom. CT AEC testing is one of the five major test areas in the Belgian CT QA protocol,^(14)^ and the authors describe a methodology to allow the independent assessment of the longitudinal and rotational aspects of the AEC system. This method involves use of the head and body CTDI phantoms (16 cm and 32 cm diameter, respectively), and a 10 cm ionization chamber. The Belgian group explains that at least three scans are required to fully assess the AEC system. Although some test results are presented, there are no suggested tolerances for metrics of dose or image quality.

Despite the paucity of guidance on QA tests for CT AEC systems, there are a number of test phantoms that have been used to assess or characterize the performance of CT AEC systems.^15^, ^16^, ^17^, ^18^, ^19^ In general terms these phantoms vary in cross‐sectional thickness along the long axis of the phantom (z‐axis of scanner) and may be elliptical or circular in cross section. Some are of a uniform material throughout, whilst others contain test details such as inserts of varying CT number, beads or wires for spatial resolution testing, and the option to insert an ion chamber for internal dose measurements. Such phantoms could be used for standardized QA testing of CT AEC systems. Ideally a CT AEC test phantom should be of elliptical construction, with varying elliptical ratios along its length and clinically representative attenuation values; however, such phantoms are not yet widely available.

Tsalafoutas et al.^(20)^ recently demonstrated that standard, nested, CT dose index (CTDI) phantoms can be simply modified in order to test the performance of CT AEC systems. Their method involved offsetting the head phantom (16 cm diameter) within the body annulus (32 cm diameter) in order to create a modified phantom in which there are three sections of differing attenuation along the z‐axis. They confirmed that this modified CTDI phantom could be used to characterize the AEC systems from two CT manufacturers. Bosmans et al.^(14)^ have also demonstrated that separate head and body CTDI phantoms can be used to assess CT AEC functionality. The usefulness of this phantom setup for CT AEC testing was also demonstrated by Iball in 2011.^(21)^

For the past 45 months our Medical Physics Department has used a modified CTDI phantom method similar to that of Tsalafoutas et al.^(20)^ to routinely test CT AEC systems as part of our CT QA program. This phantom was used as it is already available in Medical Physics Departments worldwide. The aim of this study was to demonstrate that the modified CTDI phantom method is a reproducible, sensitive, and appropriate QA test for AEC systems from four major CT manufacturers.

## II. MATERIALS AND METHODS

### A. Phantom setup

**Figure 1:**
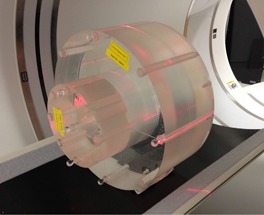
The setup of the CT AEC QA phantom on a GE Discovery 710 PET‐CT scanner.

A set of nested CTDI phantoms (Southern Scientific, Henfield, UK) was used for this study. The 32 cm diameter CTDI phantom was set up isocentrically on the clinically used CT scanner couch, in the same manner as for CTDI measurements. The 16 cm diameter head section was then offset with respect to the 32 cm annulus by half of its length, away from the gantry, thus creating a three‐part phantom consisting of the 16cm phantom (neck), 32 cm phantom (shoulders), and 32 cm phantom with 16 cm void at the center (chest). The setup can be seen in Fig. 1.

### B. Scan protocol

For each scanner a clinically used, helical, scan protocol was selected as the basis for the AEC test protocol. Wherever possible the scan protocol used the full 3D/4D AEC system, but where this was not possible, z‐axis modulation alone was selected. The clinical protocols were modified only to ensure that, wherever possible, the mA values selected by the scanner did not reach the minimum or maximum mA ratings of the X‐ray tube for any of the sections of the phantom, and that the required image slice thicknesses, typically 5 mm and 1.5 mm, were produced. Table 1 lists the scanners that were included in the study, along with a summary of the scanning parameters. On all scanners, this modified scan protocol was saved as a specific Medical Physics AEC QA protocol to ensure that the same scan and reconstruction settings were used for all future QA tests. Since the direction of the scan can influence the mA modulation pattern, the scan direction was fixed within the scan protocol to ensure that the phantom was scanned in the same direction for all subsequent tests.

**Table 1:** A summary of the scanning parameters that were used on each system

*Manufacturer*	*Model*	*Detector Configuration (mm)*	*Helical Pitch*	*AEC Setting*	*mA Range*	*Image Slice Widths (mm)*
GE	Discovery 670	1.25×16i	1.375	NI=17.54	50–440	5 & 1.25
GE	Discovery 670 Pro	1.25×16i	1.375	NI=17.54	50–440	5 & 1.25
GE	Discovery 690	1.25×32i	1.375	NI=20	20–650	5 & 1.25
GE	Discovery 710	0.625×32i	1.375	NI=17.54	50–440	5 & 1.25
GE	LightSpeed Ultra	1.25×8i	1.35	NI=11.57	50–440	5 & 1.25
GE	VCT	1.25×32i	0.984	NI=11.57	50–650	5 & 1.25
Philips	Gemini TF	32×1.25	0.906	200mAs/slice	‐	5 & 1
Siemens	Definition (Dual Source)	24×1.2/64×0.6	0.9	Ref mAs=180	‐	3 & 1.5
Siemens	Definition AS	64×0.6	0.6	Ref mAs=200	‐	5 & 1
Siemens	Definition AS+	128×0.6	0.6	Ref mAs=147	‐	5 & 1
Siemens	Definition AS+	128×0.6	0.6	Ref mAs=147	‐	5 & 1
Siemens	Definition Edge	128×0.6	0.6	Ref mAs=147	‐	5 & 1
Siemens	Emotion 16	16 x1.2	0.8	Ref mAs=120	‐	5 & 1.5
Siemens	Sensation 16	16×0.75	0.8	Ref mAs = 200	‐	5 & 1
Siemens	Sensation 16	16×0.75	0.8	Ref mAs = 200	‐	5 & 1
Siemens	Sensation 64	64×0.6	1.4	Ref mAs=200	‐	5 & 1
Siemens	Sensation 64	64×0.6	1.4	Ref mAs=200	‐	5 & 1
Siemens	Sensation 64	64×0.6	1.4	Ref mAs=200	‐	5 & 1
Siemens	Sensation Open	24×1.2	1.2	Ref mAs=150	‐	5 & 1.5
Siemens	Sensation Open	24×1.2	1.2	Ref mAs=150	‐	5 & 1.5
Siemens	Sensation Open	24×1.2	1.2	Ref mAs=150	‐	5 & 1.5
Siemens	Symbia T	2×4	1.5	Ref mAs=100	‐	5
Toshiba	Aquilion CXL	64×0.5	0.828	sd=10	80–500	5 & 1

NI=noise index, Ref mAs=quality reference mAs, sd=standard deviation.

### C. Scans performed

For all scans, the imaged length was constrained to the length of the AEC phantom. This is in contrast to Tsalafoutas et al.^(20)^ who scanned into air at both ends of the phantom. We chose to constrain the scan length so that the AEC system could select from the full range of mA values within the patient mimicking material. This also ensured that, on each scanner, the irradiated length was consistent over time.

CT AEC testing was undertaken at all acceptance and routine QA surveys over the past three years and was also undertaken on the majority of unplanned QA surveys, such as new X‐ray tube testing.

At acceptance testing, or initial, baseline testing, the phantom was scanned three times in order to determine the repeatability of the AEC system. For some more recent acceptance surveys the AEC test was repeated five times over a period of two days. Also at acceptance testing, additional scans were performed for all available AEC options — for example, 3D AEC, rotational modulation only, or z‐axis modulation alone. As such we were able to assess the functionality of separate parts of the AEC system. At all routine QA visits the phantom was scanned once on the standard scan protocol.

Tests were performed by one of three members of staff with between five and eighteen years of experience testing CT scanners.

### D. Data obtained

For all systems, the CTDIvol (mGy) and dose length product (DLP ‐ mGycm) values that were displayed postscan were recorded, along with the average mA or mAs if available. CTDIvol was not directly measured as part of the AEC test, but the accuracy of the scanner displayed CTDIvol values under a variety of scan conditions was assessed separately during the QA testing procedure. All images produced during testing were exported from the scanner and subsequently analyzed off‐site.

The scanner selected mA values were extracted from each acquired image using the DICOM Info Extractor software.^(22)^ These mA values were then plotted against the z‐axis slice position or image number in order to generate an mA modulation profile for each scan. Analysis was performed on each image, for both slice thicknesses.

**Figure 2:**
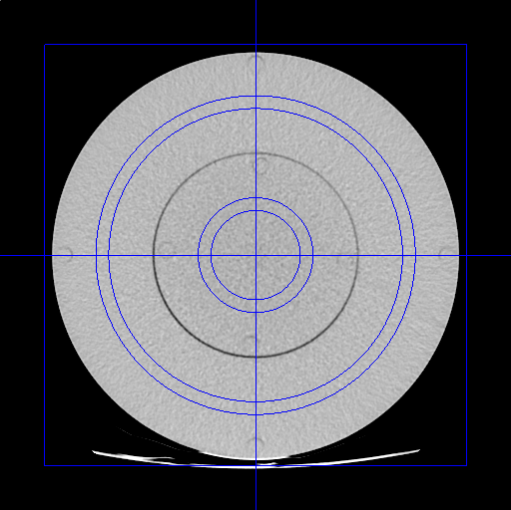
An axial image through the shoulder section of the phantom showing the two ROIs used for the image noise assessment.

In order to gather image noise data, a simple analysis tree was developed within IQWorks.^(23)^This analysis tree placed two annular regions of interest (ROIs) within the phantom; one within the 16 cm diameter section, and the second within the 32 cm diameter annulus (Fig. 2).

The ROIs were 1 cm in diameter and were positioned isocentrically with radial positions that were chosen to avoid the CTDI phantom rods and the nylon screws that secured the back plates of the phantom in place. For each region of interest the CT number and standard deviation (noise) were recorded. The standard deviation values were plotted against the z‐axis slice position in order to generate a noise profile for each scan. Noise values at the boundaries of the three sections of the phantom, where the tube current should be changing rapidly, were expected to be unrepresentative of the overall noise in the three separate phantom sections; thus, for all further noise analysis, data was only taken from images that were within the central 5 cm of each of the phantom sections. Noise values for each of the regions of interest in each of the phantom sections were averaged and these average values were further combined to produce an overall noise figure for the whole phantom.

For all routine surveys, the data obtained were compared with the baseline values for that system, which were established either when the system was acceptance tested, or when initial CT AEC tests were performed. Baseline values were only reset when the scanner had undergone major maintenance, such as replacement of the X‐ray tube, or when the CT AEC scan protocol was amended.

### E. Systems tested

Over the past 45 months the CT AEC test has been performed on 23 different scanners with a routine testing frequency of 6 or 12 months, according to the location of the scanner. These tests have been performed on systems from four manufacturers: GE Healthcare (Milwaukee, WI), Philips Medical Systems (Best, The Netherlands), Siemens Healthcare (Forcheim, Germany) and Toshiba Medical Systems (Otawara, Japan).

The breakdown of scanners by manufacturer was: GE: 6; Philips: 1; Siemens: 15; Toshiba 1. In total the CT AEC test has been performed on 115 CT QA surveys over this period. The systems tested, and the total number of surveys for each system, are shown in Table 2.

The Siemens Dual Source system was tested in both single source and dual source scan modes, thus giving 12 sets of data.

For all subsequent analysis, scanners were broadly grouped by manufacturer with the exception of Philips and Toshiba which, due to the small number of scanners in the study, were grouped together. Since the Siemens RT systems incorporate an additional carbon fiber flat couch top, these systems were separated from all other Siemens scanners to form a fourth group.

The dose data presented relate to systems that have been tested on more than one occasion; this equated to 111 data sets. Both the displayed CTDIvol and DLP were recorded from the scanner, but since these two are related via the irradiated length, which was consistent for all tests on a given scanner as it was fixed within the scan protocol, we have focused the dosimetry aspects of the results on the displayed CTDIvol.

Image noise data were available for 102 of the 111 datasets. Noise data were not available for nine surveys due to failed export of the images to storage disc.

**Table 2:** A summary of the CT scanners that were included in the study

*Manufacturer*	*Model*	*AEC System*	*Number of Times Tested*
GE	Discovery 670^a^	AutomA 3D (AutomA & SmartmA)	2
GE	Discovery 670 Pro^a^		1
GE	Discovery 690^a^		8
GE	Discovery 710^a^		1
GE	LightSpeed Ultra		5
GE	VCT		5
Philips	Gemini TF^a^	DoseRight 2.0	8
Siemens	Definition (Dual Source)		6 (12)
Siemens	Definition AS		8
Siemens	Definition AS+		3
Siemens	Definition AS+		1
Siemens	Definition Edge		1
Siemens	Emotion 16		2
Siemens	Sensation 16		7
Siemens	Sensation 16	CARE Dose 4D	5
Siemens	Sensation 64		6
Siemens	Sensation 64		7
Siemens	Sensation 64		6
Siemens	Sensation Open^b^		8
Siemens	Sensation Open^b^		6
Siemens	Sensation Open^b^		7
Siemens	Symbia T^a^		2
Toshiba	Aquilion CXL	SURE Exposure 3D	4

a ^a^ Denotes scanners that are part of PET‐CT or SPECT‐CT systems in Nuclear Medicine (NM).

b ^b^ Denotes scanners that are used in Radiotherapy (RT).

## III. RESULTS

**Figure 3:**
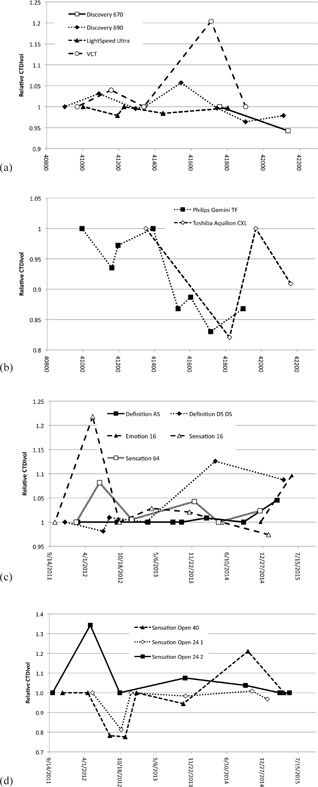
The variation in average CTDIvol values relative to baseline for: (a) GE, (b) Philips and Toshiba, (c) Siemens diagnostic scanners, and (d) Siemens RT scanners.

The CTDIvol relative to the baseline value for the GE scanners is shown in Fig. 3(a), for the Philips and Toshiba systems in Fig. 3(b), for the Siemens diagnostic scanners in Fig. 3(c), and for the Siemens RT systems in Fig. 3(d). Due to the large number of systems in the Siemens diagnostic scanners group, we have presented data for a subset of systems which includes the systems for which the greatest deviations from baseline results were found, and one system which yielded the least variation in performance.

For each manufacturer, there were deviations in CTDIvol of greater than 15% relative to the baseline value, and the maximum deviation from the baseline of 34% was observed on a Siemens Sensation Open scanner.

The percentage of CTDIvol test results that varied from baseline by more than 5%, 10%, and 15% were 23.4%, 12.6%, and 8.1%, respectively. These results, and the total number of surveys performed, are summarized for the four groups of scanners in Table 3.

Similarly, for image noise, deviations of greater than 5% relative to the baseline value were observed for each manufacturer, and the maximum deviation from the baseline of 17% was observed on a Toshiba Aquilion CXL system. For the whole dataset, the percentage of image noise results that varied from baseline by more than 2%, 5%, and 10% were 26.5%, 5.9%, and 2%, respectively.

The percentage of noise results with a deviation from baseline of more than 5% for the four groups of scanners is shown in Table 4, along with the total number of datasets analyzed.

**Figure 4:**
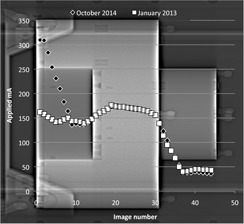
mA profiles for AEC tests on a Siemens Sensation Open system, demonstrating increased mA in the chest region of the phantom due to positioning over the dense section of the couch.

Figure 4 shows the mA profiles from one of the Siemens RT systems for surveys performed in January 2013 (new X‐ray tube) and October 2014 (routine). The mA profile from January 2013 shows low mA through the neck section, high mA through the shoulders and slightly reduced mA through the chest. In general the profile from October 2014 shows good agreement with this baseline profile, except in the chest section in which the applied mA rose to double the value obtained at baseline; this resulted in an average CTDIvol for the whole scan which was 21% higher than the baseline that was set in January 2013.

**Table 3:** A summary of the CTDIvol deviations from baseline for the four scanner groups. Value in brackets is the absolute number

*Scanner Group*	*Number CTDIvol Datasets*	*Percentage Showing Deviation* >5% *from Baseline*	*Percentage Showing Deviation* >10% *from Baseline*	*Percentage Showing Deviation* >15% *from Baseline*
GE	20	15 (3)	5 (1)	5 (1)
Philips and Toshiba	12	58 (7)	42 (5)	17 (2)
Siemens Diagnostic CT	58	16 (9)	5 (3)	2 (1)
Siemens RT	21	33 (7)	24 (5)	24 (5)

**Table 4:** A summary of the image noise deviations from baseline for the four scanner groups. Value in brackets is the absolute number

*Scanner Group*	*Number Image Noise Datasets*	*Percentage Showing Deviation* >2% *from Baseline*	*Percentage Showing Deviation* >5% *from Baseline*	*Percentage Showing Deviation* >10% *from Baseline*
GE	20	40 (8)	5 (1)	0 (0)
Philips and Toshiba	10	40 (4)	40 (4)	20 (2)
Siemens Diagnostic CT	53	21 (11)	2 (1)	0 (0)
Siemens RT	19	21 (4)	0 (0)	0 (0)

**Figure 5:**
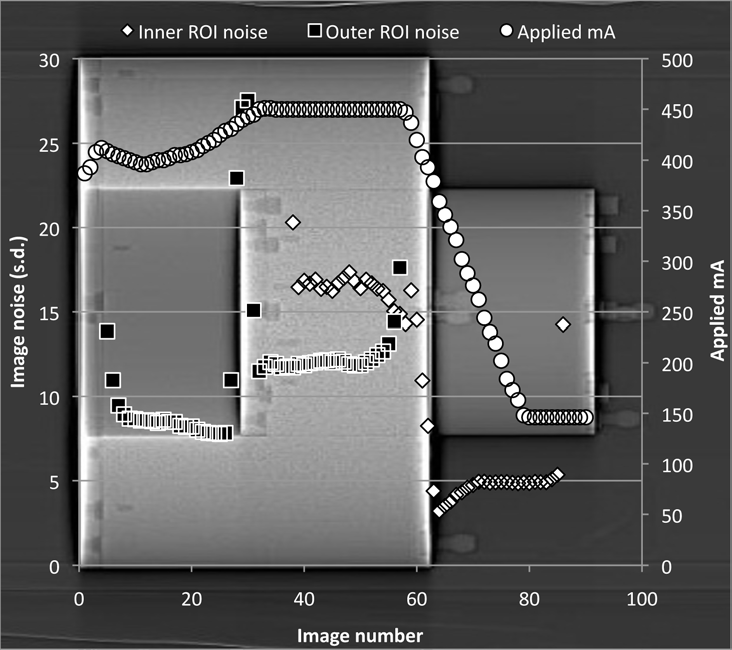
Shown is the mA profile and image noise values for a scan using z‐axis tube current modulation (ZDOM).

The mA and image noise profiles for the Philips Gemini TF PET‐CT system are shown in Fig. 5.

The mA profile shows the same overall shape as that for the Siemens Sensation Open scanner in Fig. 4, whilst the noise profile shows lowest noise in the neck section, with highest noise in the body section. It can be seen that, in the central region of each phantom section, the image noise values are broadly consistent, whilst outside of these areas the noise values are affected by the boundaries between phantom sections.

**Figure 6:**
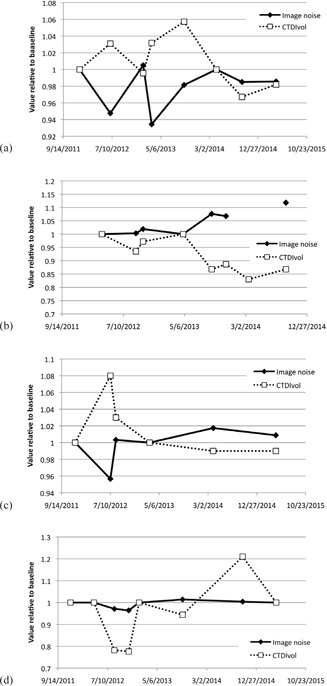
Plots of CTDIvol and global image noise relative to baseline for: (a) GE Discovery 690, (b) Philips Gemini TF, (c) Siemens Definition, and (d) Siemens Sensation Open.

Figures 6(a) to (d) show the relative CTDIvol and global phantom noise for one scanner from each of the four groups: (a) GE Discovery 690, (b) Philips Gemini, (c) Siemens Definition (single source mode), and (d) Siemens Sensation Open. The missing data point in (b) was due to a failed export of the AEC test images. This figure shows that, in the majority of cases, the changes in CTDIvol and noise are opposed, as would be expected. The only caveat to this is for the Siemens Sensation Open for which changes of ±21% in CTDIvol yielded changes of only +1% to −4% in image noise.

For Siemens scanners, we were only able to test the AEC system in full 3D/4D mode. However, for all other manufacturers we were able to test some aspects of the AEC system independently. For Philips DoseRight 2.0, it was possible to test the z‐axis modulation (ZDOM) and rotational modulation (DDOM) separately, but not together. For both GE and Toshiba, it was possible to disable rotational mA modulation and therefore test z‐axis modulation alone.

**Figure 7:**
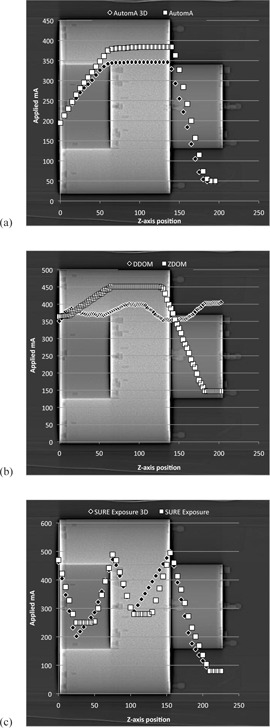
The figures demonstrate the effect of AEC setup on the obtained mA profile for: a) GE VCT, b) Philips Gemini, and c) Toshiba Aquilion CXL.

Figure 7 shows the mA profiles for the two AEC setups on the GE, Philips, and Toshiba systems: (a) GE AutomA 3D and AutomA, (b) Philips ZDOM and DDOM, and (c) Toshiba SURE Exposure 3D and SURE Exposure.

## IV. DISCUSSION

For all manufacturers deviations from baseline of >15% were observed. The majority of these large deviations resulted from incorrect phantom positioning, or incorrect protocol use.

For Siemens diagnostic systems, the CTDI deviated from baseline by more than 15% on only one occasion (Siemens Sensation 16, May 2012). When this test was performed, the rotation time for the scan was inadvertently changed from 0.5 s to 0.75 s thus allowing the scanner to deliver higher mAs values, for a given mA, which would not have been possible with the shorter rotation time of 0.5 s. Therefore, the scanner provided the requested image quality but delivered a higher CTDIvol and DLP than would have been obtained if the rotation time had not been changed.

For the Siemens RT systems all deviations >15% were attributable to the construction of the flat couch top. The iBeam Evo couch top (Elekta Ltd, Crawley, UK) has an area of increased density, and therefore reduced transmission, at the point where the head and body sections of the couch meet (Fig. 4). Wherever possible this area of the couch is avoided when positioning patients for radiotherapy treatment. The mA profiles shown in Fig. 4 demonstrate the effect of this area of increased density. The mA profile for January 2013 was obtained with the AEC phantom positioned appropriately, and the resulting mA profile is as expected. However, in October 2014 the chest section of the phantom was slightly overlaying the dense section of the couch and this additional attenuation within the beam path caused the scanner to increase the mA in this section of the phantom, thus giving an increased CTDIvol. All other >15% deviations observed in Fig. 6(d) can also be explained in this way, with the deviation being positive or negative according to whether the baseline value was obtained with the phantom away from, or overlaying, the dense area of the couch.

The Philips DoseRight 2.0 AEC system can operate in one of two modes: manual or automatic. In automatic, or learning, mode when the operator overrides the mAs suggested by the AEC system, the scanner adjusts the size of the built‐in reference phantom which it uses for patient size calculation.^(24)^ In manual mode, the AEC system uses a built‐in 33 cm diameter phantom whereas, in automatic mode, since the size of the reference phantom can change over time, the apparent size of a fixed sized standard phantom changes over time, thus causing the scanner to vary the dose given under AEC control. The AEC system on the Philips Gemini system was set to manual for the period March‐October 2012 and, for the three AEC tests that were performed, the maximum deviation from baseline was 6%. In November 2012, the AEC setup was changed to automatic mode and in the AEC tests over the following two years, the maximum deviation from baseline was 17%. Figure 6(b) shows that, over this period, CTDIvol values decreased and noise values increased. This is indicative of a change in reference phantom, or reference image quality, over time.

For the single Toshiba system in the study, the CTDI value obtained in July 2014 was 18% lower than the baseline value. The scan protocol that was used in July 2014 was not exactly the same as that used at the baseline survey in March 2013 and, as such, the July 2014 result is not directly comparable with the baseline value. Aside from this result, CTDIvol deviations from baseline were within 10%.

The sole deviation of >15% for GE scanners (VCT, March 2014) cannot be easily explained by phantom setup or protocol choice. At the time of the survey, the test was repeated three times and the same CTDIvol and DLP values were obtained on each occasion. In addition to this, the radiographer's weekly AEC test was performed and the results that were obtained were within 0.5% of the baseline values. The AEC test images from March 2014 failed to export and were deleted from the system by the radiographers on the same day. As such we were unable to assess the image noise and cannot, therefore, provide a full explanation for the high CTDIvol and DLP values that were recorded. Given that the results of the radiographer's AEC test were within tolerance, and that there was no observed change in patient doses at this time, we are unable to explain the observed deviations. The X‐ray tube on this system was replaced in September 2014 and the baseline values were reset.

Removing the deviations of >15% that can be explained by phantom setup or protocol choice, leaves only one occasion where the deviation from baseline exceeded 15%. This gives a failure rate of 0.9% (1 in 111).

Similarly for image noise, two of the six deviations of greater than 5% are attributable to incorrect protocol selection, whilst a further three are attributable to the use of automatic AEC mode on the Philips Gemini system. This leaves only one unexplained variation in image noise of greater than 5%, which equates to a failure rate of 1% (1 in 102).

Changes in measured image noise and scanner‐displayed CTDIvol values were generally opposed, as would be expected; a lower CTDIvol value should result in increased image noise. The exceptions to this were the Siemens Sensation Open systems (Fig. 6(d)). The changes in CTDIvol over time are explained by positioning of the phantom relative to the dense part of the couchtop. When the AEC phantom was positioned over the dense area of the couch, the X‐ray tube still had a sufficiently high maximum mA to allow the scanner to deliver the requested image quality; so in these cases, although there was a change in CTDIvol, there was no significant change in image noise. These results demonstrate the importance of evaluating both CTDIvol and image noise as part of a routine AEC QA test.

Based on the results of this study we would suggest that tolerances of ±15% for CTDIvol and ±5% for image noise are achievable with careful phantom setup and protocol choice. Specifically, the following conditions need to be consistent for all tests:

- The phantom must be positioned isocentrically, with the neck section offset from the shoulder section in a consistent direction.
- The phantom should be level; if necessary, the scanner mattress should be removed.
- The scan protocol used should be identical in terms of the scan, reconstruction, and AEC parameters. For Siemens systems, the CARE Dose adaptation strengths should be checked for consistency. We recommend that a dedicated AEC QA testing protocol is saved on all scanners.
- Finally, the scan direction (e.g., craniocaudal) and scan length must also be consistent.

It is also necessary to view the mA and image noise profiles relative to those obtained at baseline, as this provides spatially localized information which is not immediately available from the scanner displayed CTDIvol and DLP values. The proposed tolerances could be applied to individual points on the mA and image noise profiles. However, any small misalignment of the scan volume with respect to the AEC phantom would cause changes in mA and image noise which would be most notable at the boundaries of the phantom sections; thus a visual comparison of the mA and noise profiles is more appropriate than a point by point analysis.

AEC test results which fell outside of our recommended tolerances were raised with the radiographic staff at the time of the QA tests and in our formal report. It is unclear exactly what steps each of the service companies would take to ensure adequate performance of their AEC system. However, for Siemens scanners we are aware that the Service Engineer can undertake a “Dose Modulation (DOM) Test” within the service software, and also that there is “modulation control board” within the scanner which controls the Care Dose 4D functionality. When performance issues with Siemens AEC systems have been raised, we would expect that the performance of the modulation control board should be checked and that the DOM test should also be performed.

A reliable QA test should be both repeatable and sensitive to changes in the system performance.

For a series of Siemens diagnostic CT systems installed between December 2014 and June 2015, the AEC tests performed over the two days of acceptance testing showed maximum deviations from the mean CTDIvol values of 0.5%, 1.3%, 1.7%, and 2.1%, which shows a high level of repeatability.

The sensitivity of the AEC test to changes in the AEC setup was demonstrated on the GE and Toshiba scanners by disabling the rotational aspects of tube current modulation. The mA profiles shown in Figs. 7(a) and (c) demonstrate that deactivating the rotational tube current modulation caused a change in the mA values selected across the three sections of the phantom. For the GE scanner, higher mA values were selected in each of the phantom sections, resulting in an increase in the average CTDIvol of 8% (9.84 mGy vs. 9.07 mGy). For the Toshiba scanner the effect of the deactivation of rotational modulation was less clear. With rotational mA modulation deactivated, mA values were higher in the neck, lower in the shoulders, and higher in the chest sections, respectively, resulting in a 6% increase in CTDIvol.

Figure 7(b) shows the mA profile obtained on the Philips Gemini for rotational modulation alone. This demonstrates that the applied mA is broadly consistent through the three sections of the phantom, but that changes do occur at the section boundaries. For a helical scan, the join of the neck and shoulder phantom sections will be traversed during one rotation, and as such, the scanner will encounter a change in rotational attenuation, which the AEC system will attempt to account for by adjusting the mA. This is demonstrated by the 7%‐10% variation in mA at the phantom boundaries. These results demonstrate that, under helical scan conditions, the phantom is sensitive to rotational aspects of CT AEC systems. Although the AEC phantom itself is cylindrically symmetrical, the presence of the CT couch provides an inherent rotational variation in attenuation, which, coupled with the angular variation in attenuation experienced by the scanner as the helical scan traverses the section boundaries, provides some rotational changes in attenuation. This was confirmed by Tsalafoutas et al.,^(20)^ who showed that, for the AEC phantom, the scanner‐calculated mA values were different between the two orthogonally acquired scan projection radiographs, thus demonstrating that the scanner is discerning rotational differences in attenuation.

However, since the scanner‐displayed mA values for each slice represent an average of the rotationally varying mA values that were used during the rotation, simply using these mA values does not allow the user to fully appreciate how the mA was varied during the scan. Since we were not able to obtain the projection‐by‐projection tube current values, we were not able to fully evaluate the usefulness of this phantom for assessing the performance of rotational tube current modulation systems. It is clear, however, that in order to more robustly assess the performance of a rotational tube current modulation system, an elliptical phantom should be used.

Figures 4, 5, and 7 demonstrate the differences in the mA modulation profiles between the four manufacturers. The Philips ZDOM and Siemens mA modulation profiles are similar in shape and show a maximum‐to‐minimum mA ratio of approximately 3; whilst there is almost an eightfold variation in mA for the GE scanner. The GE AEC system is a constant noise system, which aims to keep image noise constant for all images within a scan, whilst the Philips and Siemens systems are both adequate noise systems which allow higher noise values in thicker or more attenuating parts of the scan.^(25)^ In order to obtain constant noise in each image, a constant noise AEC system must adjust the mA according to patient attenuation more rapidly than an adequate noise system. Our results demonstrate that the mA adjustment for the varying attenuation levels within the AEC phantom is more significant in the GE scanner than in the Philips and Siemens systems.

The Toshiba AEC system is also a constant noise system, but the observed mA modulation pattern (Fig. 7(c)) was markedly different from that of the GE system (Fig. 7(a)). For the scan without rotational mA modulation (Sure Exposure), the AEC delivered, albeit briefly, stabilized mA levels in the three phantom sections, with notable mA peaks at the section boundaries. However, when rotational modulation was incorporated (Sure Exposure 3D) the mA did not stabilize in any of the phantom sections. This was also observed by Tsalafoutas et al.^(20)^ It is unclear why the Toshiba AEC system should appear to perform so differently from the GE system, but this may be due to the way that the Toshiba AEC system adapts to the very abrupt attenuation variations in the phantom; such abrupt attenuation changes are unlikely to be encountered in a clinical scan. Söderberg and Gunnarson^(25)^ demonstrated similar mA modulation profiles within an anthropomorphic thorax phantom for GE and Toshiba 64 slice scanners, which provides reassurance that the abrupt changes in mA observed in this phantom are not typical of the mA variations seen in clinical use when Sure Exposure 3D is used.

CT AEC systems are controlled by both hardware (detectors and modulation control boards) and software, and a fault in, or miscalibration, of any of these could cause a significant change in the functionality of the AEC system. Given that AEC systems are employed for the vast majority of clinical scans, the correct functioning of the AEC system is of paramount importance in obtaining the clinically required image quality at the appropriate patient dose. It is therefore important that the performance of the AEC system is regularly assessed, although it may be that an annual check as part of Medical Physics QA is not sufficient, but rather that more regular testing by the users would provide on‐going assurance of the system's performance prior to clinical use of the scanner.

This study has a number of limitations. Firstly, since the AEC phantom is uniform it was not possible to assess the effect of CT AEC on CT numbers, spatial resolution or low contrast detectability. However, Rizzo et al.^(26)^ and Allen et al.^(27)^ have shown that, although image noise is affected by use of CT AEC, overall diagnostic efficacy of clinical images is unaffected. Iball et al.^(28)^ demonstrated that use of tube current modulation for helical scans of an elliptical phantom resulted in statistically significant changes in image noise, but the small changes in CT numbers and signal‐to‐noise ratio that were observed were not statistically significant. It is therefore suggested that assessment of image noise is the most important AEC‐related image quality indicator to assess as part of a quality assurance test.

Secondly, we were only able to perform CT AEC tests on one system from both Philips and Toshiba and, as such, it is difficult to have a high level of confidence in the results that were obtained. Ideally this work would extend to a greater number of scanners from these two manufacturers.

Thirdly, the nested CTDI phantoms are not available in all Medical Physics departments, as some manufacturers produce separate 16 cm and 32 cm phantoms. As a result, some Medical Physics services would not be able to perform the CT AEC test exactly as described in this paper. However, positioning the two phantoms end‐to‐end on the couch and scanning the whole length of the phantom would allow an equivalent assessment of the AEC functionality to be made.

## V. CONCLUSIONS

This study has demonstrated that routine quality assurance testing of CT AEC systems can be undertaken with a modified set of nested CTDI phantoms. Tests performed over a period of 45 months showed that, although AEC systems from four manufacturers have differing performance, the results of AEC tests in terms of CTDIvol and image noise were repeatable and showed a high level of consistency. Differences that were observed could generally be explained by incorrect phantom positioning, or incorrect scan protocol use. The phantom has been shown to be sensitive to longitudinal, rotational, and combined AEC systems and is, therefore, suitable for assessing the functionality of all CT AEC systems. Based on the results of this study, we propose that CT AEC testing should be part of all routine CT quality assurance tests, and that tolerances of ±15% for CTDIvol and ±5% for image noise are appropriate. The variation of mA and image noise, along with phantom length, should also be compared with the trends observed at baseline testing.

## COPYRIGHT

This work is licensed under a Creative Commons Attribution 3.0 Unported License.
